# Development and validation of an experience of time alone scale for borderline personality disorder

**DOI:** 10.1371/journal.pone.0217350

**Published:** 2019-05-23

**Authors:** Yvette Vardy, Nicholas J. S. Day, Brin F. S. Grenyer

**Affiliations:** Illawarra Health and Medical Research Institute and School of Psychology, University of Wollongong, Wollongong, New South Wales, Australia; Medical University of Vienna, AUSTRIA

## Abstract

**Background/Objective:**

Intolerance of aloneness is considered a core feature of borderline personality disorder (BPD) that is clinically significant, yet under-researched. This study developed a measure of aloneness for individuals with BPD.

**Method:**

Interviews investigating the experience of aloneness for BPD participants (*n* = 12) formed the basis for the development of the measure. Pilot testing then occurred with BPD participants, control participants and qualified respondents. Validity, reliability and factor analysis of an Experience of Time Alone Scale (ETAS) was conducted with BPD participants (*n* = 112) and a comparison control group (*n* = 105).

**Results:**

A three factor structure was revealed: (a) Cannot Cope Alone (α = .92), (b) Need to Escape from Others (α = .90), and (c) Consumed in Intolerable Distress (α = .88). The measure correlated significantly (*p* < .01) with the Mental Health Inventory, the Aloneness and Evocative Memory Scale, and the Hurvich Experience Inventory- Revised. Comparisons revealed highly significant differences between the BPD sample and control group on all subscales and the total score (*U* = 75.5, *p* = < .001, *r* = -.85).

**Conclusions:**

This study represents one of the first empirical examinations of a construct that has largely only been studied theoretically. This newly developed measure may contribute to diagnosis and therapy.

## Introduction

Borderline personality disorder (BPD) involves instability of interpersonal relationships, self-image, behaviours and affects [1]. Globally, personality disorder prevalence is estimated at 6% [2], but individuals with BPD can account for up to 26% of emergency presentations and 25% of inpatient admissions within Australia [3], reflecting a mental health priority area [4]. Intolerance of being alone was once a criteria for BPD in the Diagnostic and Statistical Manual of Mental Disorders [5], however, in spite of it being one of the more discriminating features, its inclusion in DSM-IV was voted down by committee members who favoured an atheoretical perspective [6]. Despite its deletion, intolerance of being alone remains a key descriptive feature for BPD [7, 8]. As revision of personality disorder criteria is still ongoing [9], intolerance of being alone remains a clinically relevant yet under-examined feature of BPD that is worthy of further study.

Psychoanalytic theorists have explored both the concept of aloneness and its relationship to borderline pathology. One explanation for the intolerance of being alone in BPD may be that individuals experience annihilation anxiety [10]. This is a traumatic anxiety based on an actual experience of danger and psychic helplessness [11], reflecting a fear of impending psychic or physical destruction [12]. Helplessness may arise post-birth, when a child experiences independent needs and yet total dependency. The dependency is thought to evoke anxiety in the infant that if not met with consistent care and the subsequent internalization of a soothing other (an introject which continues the carer’s function in their absence), is experienced as annihilation of the self [13–18]. Empirical evidence from the field of developmental psychology demonstrates this fundamental need for connection and for consistent care of caregivers, as well as the distress experienced when this care is absent or removed. Meta-analyses of well-known paradigms such as the ‘strange situation’ and the ‘still face task’ indicate that the quality of parental connection is related to the subsequent attachment style of the infant [19, 20] with implications for psychopathology [21–23] and peer relations [23, 24] in later life. Thus aloneness has been contrasted with loneliness [25], as loneliness depends on an internalized sense of another who is absent, aloneness is instead a disorganizing feeling of utter helplessness because of a failure to evoke any sense of a soothing other [26]. This annihilation anxiety may also be seen as a key component of the after-effects of trauma, neglect or abuse [27], commonly thought to be contributing factors in the etiology of BPD [28].

Fonagy draws on deficits in mentalization to understand intolerance of aloneness, and again suggests that these problems may arise from deficiencies in emotionally attuned care for the infant, including a lack of contingent and marked mirroring of the child’s internal states [29]. Problems in mentalizing, or in the ability to think about one’s own or others’ mental states, is hypothesized to lead to a sense of emptiness and isolation, a depreciated sense of connection, discontinuity between past and present [30] and ultimately a diminished sense of identity. Furthermore, with chronic inaccurate reflection or maltreatment from a care-giver during development, Fonagy, et al [31] postulate that a malevolent and critical ‘alien self’ can develop and become internalized within the child, persisting into adulthood. When alone this alien self can attack the individual from within and thus creates intolerable distress from internal persecution. Suicide, self-harm and externalization of this difficult part of the self become a means of escape. A failure in secondary representation, or mentalization, akin to Meares, et al [32] loss of self-awareness or of the stream of consciousness in BPD, would result in these punitive thoughts being experienced as real, intense and immediate, rather than representations of reality that are one step removed. Thus, both deficits in mentalization and the resulting immediate experience of punitive internal states may result in distressing aloneness.

Adler and Buie [33] argued this painful aloneness is at the core of BPD and explains clingy desperate behaviours, rage when needs are unmet, self-hatred when fears of rejection arise, and impulsive attempts to avoid the feeling via drinking, sexual activity, suicidal and self-harming behaviours [34, 35]. Similarly, intolerance of aloneness has been argued to be a key feature underpinning most if not all DSM criteria for BPD [36]. As such, the frequency and intensity of aloneness may reflect the severity of BPD, and therefore it is of major importance in treatment [33, 37].

Given the diagnostic relevance of this experience in BPD, it is surprising that only one empirical examination of aloneness has been conducted. By adding items drawn from psychoanalytic traditions to the University of California, Los Angeles (UCLA) Loneliness Scale [38], Richman and Sokolove [39] created the Aloneness and Evocative Memory Scale (AEMS). This scale focuses on emptiness, inner deadness, hollowness, the inability to self-soothe and the inability to recall comforting images. Using a BPD sample and a neurotic sample, the authors found that the experience of aloneness was the strongest predictor of BPD diagnosis. When aloneness was combined with memory quotient and level of depression, use of the scale could discriminate between 87.5% (35 of 40) of borderline and neurotic patients and the authors concluded that aloneness represents a defining feature of BPD. While not explicitly examining ‘aloneness’, recent empirical research using network analyses on the related concepts of ‘loneliness’ and ‘abandonment avoidance’ both highlight that the experience of being alone is intolerable for many individuals with BPD and that this is a defining feature of the disorder [40, 41].

More empirical research is needed regarding intolerance of aloneness in BPD. Consequently, this paper first aimed to investigate the actual experience of time alone for individuals with BPD. From this, the study aimed to develop a measure that reflects this described experience, in contrast to the AEMS [39] that evolved from an existing loneliness scale and was based on theoretical notions only. Finally, the study then aimed to evaluate the developed measure in terms of consistency and reliability. If considered a core feature of BPD, understanding and being able to measure the nature of the experience of being alone may give clinicians a direct measure of the severity of the illness. It may also provide a measure of the integrity of the self, an important insight into the capacity to soothe and regulate the self and a vital way to empathize with this painful experience. Given therapeutic relationships are self-evidently not in the context of being alone, a scale assessing this state may also yield important clinical information about specific problems and deficits that are difficult for the client to recall in an interpersonal environment.

## Study 1: Investigation into the experience of aloneness in BPD

### Aims and hypotheses

The aim of this study is to investigate the experience of time alone for individuals with BPD. It is hypothesized that participants will report an overall negative experience of time alone characterised by distressing internal experiences. Whilst the exact characterisation is unknown and provides the rationale for this qualitative study, it is expected time alone may be described as frightening in some way and aversive, and perhaps even intolerable, overwhelming or annihilating. It is expected that participants will report utilizing a range of strategies to avoid this experience.

### Method

#### Participants

Twelve women with a primary diagnosis of BPD attending the Affect Regulation Clinic were invited and agreed to participate in consultation with their treating clinician. The clinic, specializing in providing evidence-based psychological therapy for those with borderline personality disorder [36], operates in the local health service under a university-health service partnership [42]. Six of the participants with BPD had been assessed as having comorbid diagnoses of mood and anxiety disorders, and one participant also identified as engaging in alcohol abuse. Diagnosis was initially established by their treating psychiatrist or clinical psychologist, and confirmed with the SCID-II [43]. This number of participants is satisfactory for qualitative analysis [44, 45]. Participants’ age ranged from 19 to 56 (*M* = 36.3 years) and half were in a romantic relationship. All individuals agreed to participate following informed written consent procedures approved by the University of Wollongong’s Human Research Ethics Committee (HE2006/008).

#### Procedure

Interviews were conducted face-to-face and audiotaped with consent from participants. Interviews involved five open ended questions, carefully constructed so as not to influence the nature of the responses. These were as follows:

1. *Generally speaking*, *how do you feel about time alone*?
2. *What influences your experience of time alone*?
3. *What do you do when you’re alone*? *How does this differ in quality (feel) or content (the things you do) when around others*?
4. *Describe a typical experience of a time when you were alone*. *Try to get as detailed as possible so I really understand exactly how it felt for you and what you were thinking*.
5. *Tell me about any times and ways you’ve tried to avoid the experience of time alone*. *Describe any times and ways you’ve deliberately sought the experience of time alone*.

For complete transcripts and a more detailed account of the procedures followed, see Vardy [46].

#### Analysis

Phenomenological analysis followed the procedure outlined by Giorgi [47] which assumes that if there is a common phenomenon of aloneness that people with BPD experience, then each individual with BPD would convey elements of that experience in their interview. To ensure reliability and clinical utility two researchers independently identified ‘meaning units’ of aloneness from the scripts [48]. The researchers were both doctoral level clinical psychologists working within a personality disorders program. Through collaboration these units were synthesized into ‘consensus categories’ and further again into overall ‘themes’ that expressed the underlying psychological phenomena being described. For instance, the meaning units of: ‘more thoughts, worries, negative thoughts, constant thoughts’, ‘hears voices when alone’, ‘rumination increase’ and ‘busy mind when alone’ where all grouped together into one consensus category, as they were all viewed as expressing the same psychological phenomenon. This consensus category was labelled ‘intrusive negative thoughts’. The process of grouping meaning units into consensus categories helped to clarify when the different language or labels used by the two independent raters shared the same underlying psychological characteristics.

The categories were condensed further into themes based on function. Both researchers collaborated and used imaginal variation to reflect on when and how each category operated, and whether any tended to co-occur. For example, “alcohol/substances to escape or avoid”, and “sleep/dissociate as avoidant behaviour” were both considered part of the same theme “maladaptive attempts to escape distress” as they both occurred at the same times, and were used by participants in the same ways to manage time alone. The researchers then independently calculated the number of participants they endorsed as experiencing each theme and the Spearman’s Rank Reliability Coefficient highlighted strong, positive agreement between the two sets of ratings (*r* = 0.78).

### Results

Tables 1 and 2 display the themes and their latent meanings identified in the interviews. These themes encapsulate the essence of aversive (negative) and craved (positive) experiences of time alone for the twelve participants. As shown in Table 1, nine overarching themes were identified for a negative experience alone, consisting of (a) Intrusive negative thoughts; (b) Insecurity, conflict and indecision; (c) Self-recrimination; (d) Inability to control escalating panic and rage; (e) Maladaptive attempts to escape distress; (f) Avoidance of aloneness by structuring and filling time; (g) Reassurance and direction from others; (h) Lethargy, inactivity and amotivation; and, (i) Feelings of depression and disconnection.

**Table 1:** Participants’ negative experience of time alone (N = 12).

Theme	Latent meaning
**Intrusive negative thoughts**	Increased or constant rumination, worries. Increased suicidal thoughts. Bombarding thoughts.
**Insecurity, conflict and indecision**	Lack of confidence and direction. Fears of doing the wrong thing. Self-doubts and inner conflict result in inability to initiate or persist with activity. Indecision and cognitive battles.
**Self-recrimination**	Self-hate and negative self-judgement. Shame, guilt and regret. Thoughts ranging from ‘I should be able to cope’, ‘I should never have done that’ to ‘I hate myself’, ‘I’m hopeless’.
**Inability to control escalating panic and rage**	Increased distress and frustration. Cascading emotions. Inability to regulate emotions or end episode. Rage, panic and loss of control. Annihilation.
**Maladaptive attempts to escape distress**	Self-harm, alcohol, drugs, sleep, dissociation, binge eating or suicidal ideation.
**Avoidance of aloneness by structuring and filling time**	Pre-planning of activities to avoid silence and distress. Pets, exercise, radio, TV, hobbies, company helpful. Easier to structure day when mood is better.
**Reassurance and direction from others**	Others sought out for reassurance. Others needed for decisions, motivation.
**Lethargy, inactivity and amotivation**	Feeling burdened and drained, consumed in lethargy. Simple tasks enormously effortful. Inactivity, anhedonia and dread.
**Feelings of depression and disconnection**	Feeling sad, lonely, rejected, unloved and hopeless.

**Table 2:** Participants’ positive experience of time alone (N = 12).

Theme	Latent meaning
**Freedom without compromise**	Free from feelings of obligation and from the demands of others. Can focus on self without sacrifice.
**Settled and in touch with self**	Soothing, recharging. Freedom from intrusion and distraction of others needed to get in touch with self. Full relaxation prevented by others.
**Feelings of inadequacy insecurity and rejection fears around others**	Feelings of insecurity, alienation and social inadequacy around others. Fears of judgement and rejection.
**Effortful and self-sacrificing relating**	Relating experienced as demanding. Incapacity to focus on self, given strong efforts needed to please others and communicate.
**Invasion of senses and boundaries by others**	Invasion of senses and boundaries by others. Overwhelmed and intruded upon.
**Overwhelming stress and anger around others**	Escalating agitation and irritability around others, Frustration, stress and rage.
**Desperate need to escape from others**	Urgent need to escape from others to ease discomfort. Disconnection the only means of managing distress.

Table 2 shows the seven themes identified for a positive experience alone arising from difficulties relating, namely (a) Freedom without compromise; (b) Settled and in touch with self; (c) Feelings of inadequacy, insecurity and rejection fears around others; (d) Effortful and self-sacrificing relating; (e) Invasion of senses and boundaries by others; (f) Overwhelming stress and anger around others; and, (g) Desperate need to escape from others.

Some participants described feelings of being continually uneasy and restless both alone and with others and a sense that inner peace was impossible.

## Study 2: Development, validity and reliability of a self-report measure of the experience of time alone

### Aims and hypotheses

First, this study aims to develop a measure that reflects the experience of being alone for individuals with BPD following from the results of the previous study. Second, this study aims to evaluate the developed measure in terms of validity and reliability. It is hypothesized that scores on the developed measure will be significantly higher for participants with BPD compared to healthy controls. Furthermore, that higher ratings of aloneness will significantly correlate with psychopathology, higher ratings of annihilation anxiety, and higher scores on a previous measure of aloneness which includes more failures in evocative memory.

### Item development and pilot testing

Preliminary work was undertaken to design the items for a brief self-report scale to assess the nature and severity of the experience of time alone for people with BPD. The results from Study 1 were used to inform the development of the instrument.

Items were generated for each theme derived in Study 1, including some negatively weighted items. Language in the original interviews was used whenever possible to ensure the closest fit with the essential experience. In collaboration with the research team, items were selected that most clearly expressed a single idea without ambiguity. A four part response format was chosen for simplicity and ease of use (‘Not at all’, ‘A little bit’, ‘A moderate amount’, ‘A great deal’), mirroring the response format of Richman [49] in the Aloneness and Evocative Memory Scale. Items were placed in a random order so that similar items were separated and the reverse-scored items were spread throughout the scale to minimize response bias. Sixty two items underwent pilot testing and these reflected both the themes describing the experience of time alone and those that depicted the challenging nature of time with others.

#### Stage 1: Pilot testing with BPD participants

Two female participants, aged 33 and 23 years old, both diagnosed with BPD and meeting the DSM-IV criteria according to the SCID-II [43] agreed to participate in the pilot testing. Neither participant took part in the original study and both were receiving treatment from a local health service. Based on feedback from these participants, revisions were made to various items regarding ease of understanding and clarity of items and one item, “I need time alone to be my true self”, was deleted due to reported trouble with identity and in understanding what a true self would be. Additionally, both participants raised issues with the “Time with Others” items, because their experience varied greatly depending on who the “others” were. The Time with Others section was therefore reviewed and modified in an attempt to make items clearer. Overall, participants reported that they found the questions clear, easy to understand and that the scale covered all the important areas of their experience of time alone.

#### Stage 2: Pilot testing with control participants

Five healthy control participants, two males aged 35 and 63, and three females aged 32, 34 and 34 agreed to participate in pilot testing. Feedback in general was of a positive nature, and no modifications were made to items depicting time alone. However items describing time with other people continued to be reported as ambiguous or difficult to answer. Consequently, items were converted into statements depicting the positive experience of time alone once away from others. For example “I find myself getting edgy and irritable around others” was converted into “I need to have time alone because I get irritable and edgy around others”. Those with the clearest wording were selected for inclusion in the scale, also ensuring each theme from the original study was represented by at least one item.

#### Stage 3: Pilot testing with experienced and qualified clinicians

Six health professionals (50% female) with a minimum experience of 10 years (mean = 16, *SD* = 4.18, range 10 to 20 years) in the treatment of BPD agreed to review the aloneness scale. These participants were clinical psychologists (*n* = 3), a research psychologist (*n* = 1), an occupational therapist (*n* = 1) and one nurse (*n* = 1). Respondents were asked to rate each item on the basis of clarity (ranging from ‘not clear’, ‘somewhat clear’, ‘quite clear’ and ‘highly clear’) and clinical relevance (ranging from ‘not relevant’, ‘somewhat relevant’, ‘quite relevant’ and ‘highly relevant’) as recommended by Davis [50]. The content validity index (I-CVI) was calculated for each item on the basis of these expert ratings. This was done by computing the number of experts scoring each item as either ‘quite’ or ‘highly’ (dichotomizing the ratings into clear and not clear, or relevant and not relevant), divided by the total number of experts. I-CVI’s ranged from .50–1.0 for clarity and .67–1.0 for relevance. As specified by Lynn [51], acceptable level of agreement for six or more judges’ I-CVI ratings is to be no lower than 0.78. All those with an index score below 0.78 were modified until they met criteria or deleted, any that had an I-CVI below 1.0 were improved wherever possible.

Items retained at the end of pilot testing are presented in Table 3 as grouped into their themes. These modifications resulted in 58 items remaining in the scale.

**Table 3:** Items grouped according to themes from preliminary study.

Items for each theme
Intrusive negative thoughts
When I am alone my mind is so busy it does not stop
When I am alone I think more about suicide
When I am alone I stress about my interactions with others
When I am alone I hear voices inside my head
When I am alone my mind becomes filled with negative thoughts about the past
Alone I can choose not to think about issues that are bothering me and get on with other things (Reverse scored)
Insecurity, conflict and indecision
I can not settle into an activity when I am alone
When I am alone I can not work out what to do with myself
When I am alone I wish someone was there to tell me what to do
When I am alone I worry that I am not thinking right
When I am alone I argue and battle with myself in my head
Self-recrimination
When I am alone I dwell on things I have done wrong
When I am alone I am very critical of myself
When I am alone I enjoy pampering and doing nice things for myself (Reverse scored)
Inability to control escalating panic and rage
When I am alone I feel so abandoned I will desperately seek contact with other people
I feel myself getting anxious when I am alone
I can feel a sense of inner peace and contentment when I am alone (Reverse scored)
Alone my mood spirals downwards and I can not stop it
When I am alone I get full of rage
I stay distressed alone until someone else can help me feel better
Maladaptive attempts to escape distress
I dissociate/space out to avoid time alone
When I am alone I eat too much
I get so upset when I am alone that I hurt myself
I sleep to avoid being alone
I need medication or alcohol/drugs to help me cope with my distress when I am alone
Alone I am able to calm myself down if I am upset (Reverse scored)
Avoidance of aloneness by structuring and filling time
Silence is scary when I am alone
When I am alone I structure my day so I am not left doing nothing
I seek out others to avoid being alone
I need the TV, radio or music on to fill the silence when I am alone
To cope alone I have to keep myself busy with activities
When I am alone I use drugs or alcohol to escape for a while
I can enjoy doing activities by myself
Lethargy, inactivity and amotivation
When I am alone I feel motivated to do things that I enjoy (Reverse scored)
Alone I feel overwhelmed by simple tasks and have to push myself to do them
When I am alone I sit and do nothing for hours
Feelings of depression and disconnection
When I am alone I still know that people care (Reverse scored)
When I am alone I crave having a deep personal connection with someone
I feel hopeless about my life when I am alone
When I am alone I feel lonely and wish for company
When I am alone I still feel my life has meaning and purpose (Reverse scored)
Alone I isolate and hide away from the world
Reassurance and direction from others
When I am alone I wish someone was with me to help me feel OK
When I am alone I wish someone was there to motivate me
Feelings of inadequacy, insecurity and rejection fears around others
Time alone is freedom from the worry that I will say or do the wrong thing around others
It is a relief to be alone because I do not have to maintain a false self or mask to conceal the real me
I avoid being around others because I feel like I do not fit in
Freedom without compromise
Time alone is relief from feeling self-conscious around others
When I am alone I enjoy the freedom to do what I want, when I want
I need time alone to escape from the pressure of other people’s expectations
Settled and in touch with self
When I am alone I enjoy the chance to relax and be at peace
I need time alone to work out how I feel about things
I need time alone to unwind and de-stress
Effortful and self-sacrificing relating
I need time alone because I sacrifice my needs around others
Time alone is relief from the effort it takes to relate to others
Invasion of senses and boundaries by others
It is a relief to be on my own because I find it too intense around others
Overwhelming stress and anger around others
I need to have time alone because I get irritable and edgy around others
Desperate need to escape from others
I need to escape and be by myself to avoid being totally overwhelmed by others

After the pilot testing and subsequent modification, this study aimed to demonstrate the reliability and validity of the developed measure.

### Participants

**Fig 1:**
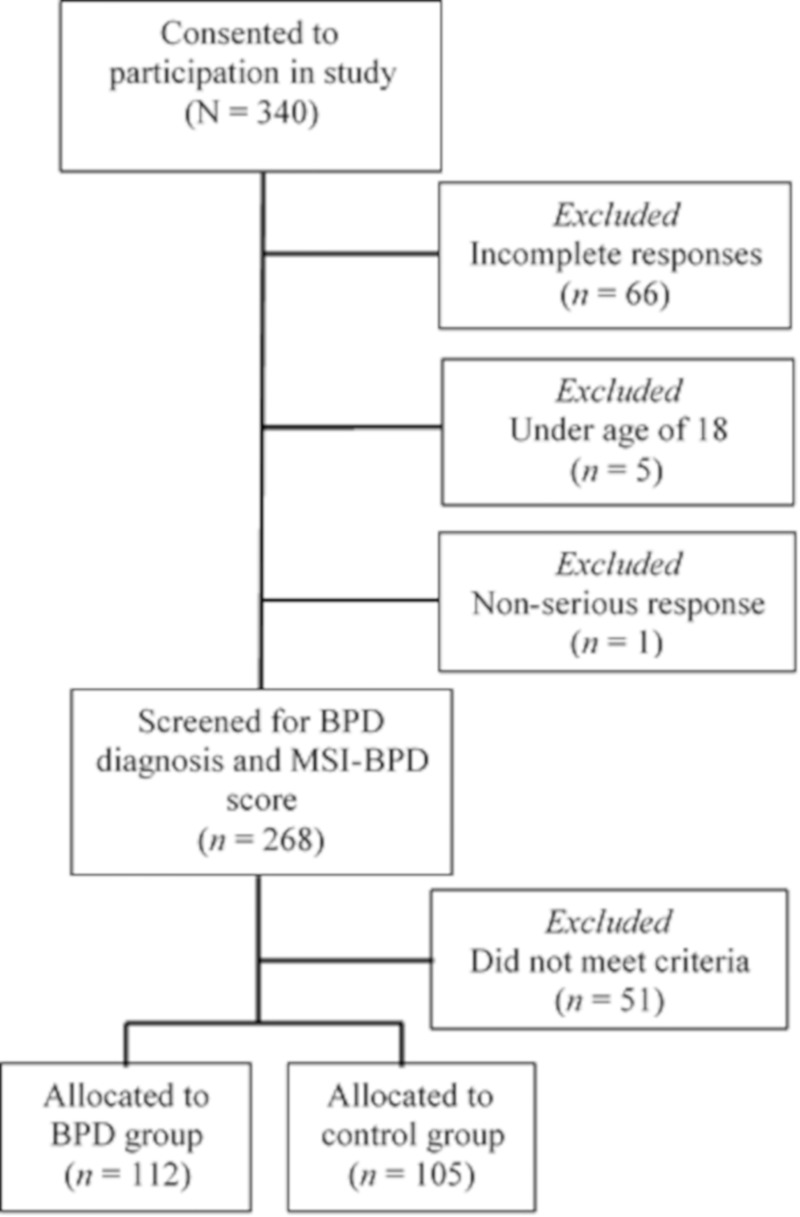
Participant flowchart. Note. BPD = Borderline personality disorder. MSI-BPD = McLean Screening Instrument for BPD.

Participants were recruited online via psychology related email lists, links on mental health websites and from BPD Facebook pages. All individuals agreed to participate following informed written consent procedures approved by the University of Wollongong’s Human Research Ethics Committee (HE2006/192). This data collection strategy via online platforms has been found to be both effective and reliable [52]. Inclusion criteria for the study were, (a) minimum age of 18; (b) completion of the survey–incomplete responses were assumed to indicate withdrawal; and (c) instances of response bias (e.g. responding the same to all items regardless of weighting). The participants were sorted into two groups on the basis of their response to the question “Have you been diagnosed with borderline personality disorder?” and their score on the McLean Screening Instrument for BPD (MSI-BPD) [53]. All respondents self-declaring a diagnosis of BPD and with a score of seven or more on the MSI-BPD were allocated to the BPD group (*n* = 112). Respondents with no reported diagnosis of BPD and with a score of four or less on the MSI-BPD were allocated to the control group (*n* = 105). Participants who did not meet either criteria were excluded from analysis (*n* = 51). See Fig 1 for this screening and group allocation process.

### Measures

#### McLean Screening Instrument–BPD (MSI-BPD)

The MSI-BPD [53] is a 10-item scale, with a Yes/No response format designed to reflect the nine DSM-IV criteria for BPD. This scale demonstrated very good internal consistency (α = .95, *n* = 217). A cut-off score of seven out of ten endorsed items has been shown to yield both good sensitivity (.81 correctly identified cases) and good specificity (.85 correctly identified non-cases) [53].

#### Hurvich Experience Inventory–Revised (HEI-R)

The HEI-R [54] is a 30-item self-report measure designed to assess annihilation anxiety, with a four-part response format of ‘Never’, ‘Not very often’, ‘Often’ and ‘Very often’. Internal consistency was demonstrated to be excellent (α = .98, *n* = 217). Content validity has been established via expert ratings and factor analysis revealed a six factor solution accounting for 54.3% of the variance, with factor loadings ranging from .40 to .81. Scores were found to significantly differ between non-clinical and clinical groups (at *p* < .001) [55]

#### Aloneness and Evocative Memory Scale (AEMS)

The AEMS [39] consists of two subscales that form a 33-item self-report scale, with answers ranging from ‘Not at all’, ‘A little bit’, ‘A moderate amount’ and ‘A great deal’. Factor 1 aloneness subscale items demonstrated an excellent Cronbach's alpha of α = .97 (*n* = 217), and the factor 2 evocative memory subscale had a very high Cronbach's alpha of α = .88 (*n* = 217).

#### Mental Health Inventory-5 (MHI-5)

The MHI-5 [56] is a five item scale that measures five dimensions (anxiety, depression, positive affect, loss of behavioural or emotional control, and psychological well-being). The MHI-5 forms the Mental Health Scale from the Medical Outcomes Study Short Form Health Survey [57]. Consistent with previous research, scores on the MHI-5 can be linear transformed to a scale of 0 to 100 [58]. Higher scores are indicative of better mental health. The MHI-5 demonstrated strong internal consistency (α = .84, *n* = 217).

#### Demographic questionnaire

Participants were asked for general descriptive information about age, gender, education, employment status, living arrangements, children and relationship status. Individuals were asked whether they had ever been diagnosed with BPD and whether they had any other current mental illnesses.

### Data screening

Data screening of the BPD sample was conducted prior to running the main analyses. Z scores were calculated to assess for univariate outliers, which were defined at +/- 3.29 standard deviations from the mean [59]. Scores ranged from z = .76 to z = 3.16 in the sample, and as such no data were excluded. Mahalanobis distance was also calculated to assess for multivariate outliers in the BPD sample, using the chi-square cut off of p < .001 for 57 items (degrees of freedom). The maximum distance 80.69 (M = 56.49, SD = 10.26) was below the critical value of 95.75, thus no data were excluded from the BPD sample. Data screening of control participant responses were also conducted. Analyses revealed a response bias to the ‘not at all’ category (reflecting comfort with time alone), and as such those who scored themselves as ‘a moderate amount’ or ‘a great deal’ were classed as outliers with z-scores above 3.29. Additionally, examination using the Kolomogorov-Smirnov text revealed that item responses were not normally distributed in either group (p <. 001). The response distribution in the BPD sample showed individuals responded most to the ‘a great deal’ category and least to the ‘not at all’ category, and the control sample largely demonstrated the opposite pattern. These differing response tendencies actually indicated that the items performed as expected, successfully reflecting the prototypical experiences of these samples and, consequently, no individual’s data were removed despite the outliers in the control sample. Visual inspection of the means obtained by both the BPD and control groups indicated that all but one item ‘I need time alone to unwind and de-stress’ performed as expected and hence this item was removed, leaving 57 items.

### Statistical analyses

Where data was not normally distributed, non-parametric tests were utilized for between-groups comparisons. As factor analysis is typically considered robust enough to handle deviations in normality [59] no data transformations were required for this test. Chi-square and *t*-tests were used to examine demographic differences, and differences between other measures (i.e. MHI-5, AEMS). Principle Axis Factoring with promax rotation was used to investigate the latent structure of the experience of time alone along with the association the current items have with this latent structure [60]. The items retained after factor analysis formed the scale. The scale was then subjected to correlation analysis with theoretically relevant measures (i.e. MHI-5, AEMS) to demonstrate validity. Finally, the Mann Whitney *U* test was utilized to test for differences between samples (BPD vs. control) for the developed measure.

### Results

Demographic details for both BPD and control groups are presented in Table 4. The majority (*n* = 94, 83.9%) of BPD participants identified that they were suffering from at least one co-morbid disorder, and given the common nature of such co-morbidities, no individual was excluded on this basis. Seven control participants indicated that they were currently suffering from a mental illness, however no significant difference was evident in their MHI-5 scores (*M* = 23.00, *SD* = 3.06) compared to the rest of the control sample (*M* = 24.78 *SD* = 3.07), *t*(103) = -1.48, *p* = .142 (two-tailed), therefore they were not excluded from the analyses.

**Table 4:** Demographic information for BPD and control groups.

	BPD (*n* = 112)	Control (*n* = 105)	Statistical comparison
Female	104 (92.86%)	87 (82.86%)	χ^*2*^ (1) = 5.14, *p* = .04
Mean Age (*SD*)	31.53 (11.21)	43.51 (13.31)	*t* (203.76) = -7.34, *p* < .001
Education (highest)			χ^*2*^ (1) = 34.14.61, *p* < .001
High school or less	58 (26.7%)	15 (6.9%)	
Post high school	54 (24.9%)	90 (41.5%)	
Employment			χ^*2*^ (2) = 43.88, *p* < .001
Full-time	27 (24.1%)	54 (51.4%)	
Part-time/casual	30 (26.8%)	42 (40%)	
Unemployed	55 (49.1%)	9 (8.6%)	
Not in relationship*	69 (61.6%)	25 (23.8%)	χ^*2*^ (1) = 31.53, *p* < .001
Do not have children	87 (77.7%)	45 (42.9%)	χ^*2*^ (1) = 27.58, *p* < .001
Live alone	34 (30.4%)	17 (16.2%)	χ^*2*^ (1) = 6.04, *p* = .01

Note.

*A relationship was defined as longer than 6 months

Means and standard deviations for the questionnaires administered in the battery are presented in Table 5. Differences between groups on all measures were significant.

**Table 5:** Group means, standard deviations and t-tests for the MHI-5, the AEMS subscales and the HEI-R.

	BPD (*n* = 112) Mean (*SD*)	Control (*n* = 105) Mean (*SD*)	Statistical comparison
MHI-5	43.83 (15.06)	79.99 (10.03)	*t* (215) = -21.744, *p* < .001
AEMS–Aloneness	70.72 (11.66)	34.35 (10.18)	*t* (213.93) = 24.519, *p* < .001
AEMS–Evocative	26.47 (5.84)	15.95 (4.61)	*t* (215) = 14.670, *p* < .001
HEI-R	89.61 (14.68)	40.14 (7.73)	*t* (215) = 30.765, *p* < .001

*Note*. *SD* = Standard Deviation, MHI-5 = Mental Health Inventory-5, AEMS = Aloneness and Evocative Memory Scale, HEI-R = *Hurvich Experience Inventory–Revised*

### Factor structure

The 57 prospective items retained after pilot testing and data screening were subjected to principal axis factoring with promax rotation utilizing the BPD sample data. The Kaiser-Meyer-Olkin measure of sampling adequacy (.80) indicated the data were suitable for factor analysis, as did Bartlett’s Test of Sphericity (*p* < .001). Horn’s parallel analysis [61] was conducted using SPSS syntax proposed by O'Connor [62] to identify the number of factors within the data, as this has been identified as the most reliable method [63]. Three factors were identified with eigenvalues of 13.41, 7.85 and 2.98 respectively. Items with factor loadings of less than .50 were suppressed (*n* = 20). Factor 1 loaded highly on 15 items, Factor 2 on 10 items and Factor 3 on 12 items, as displayed in Table 6. These factors accounted for around 41.11% of the variance in the questionnaire data. Factor 1 was labeled *Cannot Cope Alone*, depicting a need for another person in order to function and feel comfortable, the rising anger and anxiety about being left alone, and the use of alcohol and sleep to avoid the feeling of being alone. Factor 2 was labeled *Need to Escape from Others*, representing the need for time alone to escape the intrusion and demands of relating, and to escape from the negative emotions (e.g. self-consciousness) associated with being around others. Factor 3 was labeled *Consumed in Intolerable Distress*, depicting the increasing and all-consuming nature of distress when alone, in which hopelessness prevailed, and thoughts of self-harm and suicide dominated. Factor correlations revealed factor 1 and 3 to be significantly correlated *r* = .58, *p <* .001. Factor 2 did not correlate significantly with either of the other two factors and may therefore tap into independent underlying psychological characteristics. A total of 37 items across the three factors were retained after these analyses.

**Table 6:** Final items of the experience of time alone scale as grouped by its factors.

	Cannot Cope Alone (Factor 1)	Need to Escape from Others (Factor 2)	Consumed in Intolerable Distress (Factor 3)
1. When I am alone I feel lonely and wish for company	**.81**	-.16	-.23
2. I seek out others to avoid being alone	**.77**	-.15	-.20
3. When I am alone I wish someone was with me to help me feel OK	**.75**	-.24	-.09
4. When I am alone I wish someone was there to tell me what to do	**.75**	.02	-.01
5. When I am alone I feel so abandoned I will desperately seek contact with other people	**.75**	-.12	-.08
6. I feel myself getting anxious when I am alone	**.73**	-.10	.01
7. Silence is scary when I am alone	**.69**	-.05	.08
8. When I am alone I cannot work out what to do with myself	**.63**	.14	.13
9. I sleep to avoid being alone	**.63**	-.06	-.13
10. When I am alone I get full of rage	**.61**	.24	.06
11. When I am alone I wish someone was there to motivate me	**.59**	-.03	-.11
12. Alone I feel overwhelmed by simple tasks and have to push myself to do them	**.57**	.14	.16
13. I stay distressed alone until someone else can help me feel better	**.54**	-.04	.12
14. I need medication or alcohol/drugs to help me cope with my distress when I am alone	**.53**	.03	.08
15. I need to escape and be by myself to avoid being totally overwhelmed by others	.06	**.86**	.01
16. Time alone is freedom from the worry that I will say or do the wrong thing around others	.22	**.81**	-.15
17. It is a relief to be alone because I do not have to maintain a false self or mask to conceal the real me	-.18	**.76**	.19
18. Time alone is relief from the effort it takes to relate to others	-.21	**.72**	.13
19. I need to have time alone because I get irritable and edgy around others	-.12	**.69**	.16
20. I avoid being around others because I feel like I do not fit in	.09	**.68**	.10
21. I need time alone to escape from the pressure of other people’s expectations	-.10	**.60**	.13
22. *Alone I can choose not to think about issues that are bothering me and get on with other things	-.30	-.16	**.77**
23. I get so upset when I am alone that I hurt myself	-.09	.03	**.74**
24. *When I am alone I still feel my life has meaning and purpose	-.14	.01	**.67**
25. When I am alone I am very critical of myself	.12	.16	**.65**
26. *When I am alone I feel motivated to do things that I enjoy	-.12	-.07	**.63**
27. When I am alone my mind becomes filled with negative thoughts about the past	.13	.18	**.57**
28. *When I am alone I still know that people care	-.07	.06	**.56**
29. *When I am alone I enjoy pampering and doing nice things for myself	-.11	-.14	**.56**
30. When I am alone I think more about suicide	.17	.07	**.56**
31. I feel hopeless about my life when I am alone	.29	.07	**.55**
32. *I can feel a sense of inner peace and contentment when I am alone	.14	-.31	**.54**
33. When I am alone I dwell on things I have done wrong	.06	.14	**.53**

*Note*. Asterisks (*) indicate reverse scored items. Bolded items indicate items included in the scale.

### Reliability analyses

Factor 1 had an alpha coefficient of α = .92. Corrected item-total correlations ranged from .50 to .75 which classifies it as ‘very good’ [64]. Inter-item correlations ranged from .18 to .69. The lowest correlation was below the minimum *r* of .20 as specified by Ferketich [65] and was between the items ‘Alone I’m overwhelmed by simple tasks and have to push myself to do them’ and ‘When I’m alone I crave a deep personal connection with someone’. The latter item was removed from the scale because it affected alpha less and was conceptually similar to other items. The final coefficient alpha for Factor 1: Cannot Cope Alone (14 items) was α = .92 which is classified as ‘excellent’ [66].

Factor 2 alpha coefficient increased from α = .92 to α = .93 when the item ‘I need time alone to work out how I feel about things’ was removed from the scale, due to weak loading. Four items correlated above the recommended cut-off point of .70 [65] to the item ‘I need to escape and be by myself to avoid being totally overwhelmed by others’, so two items were removed (‘It is a relief to be on my own because I find it too intense around others’ and ‘Time alone is a relief from feeling self-conscious around others’). The other two items (‘Time alone is freedom from the worry that I will say or do the wrong thing around others’ and ‘It is a relief to be alone because I do not have to maintain a false self or mask to conceal the real me’) were retained, as they were thought to reflect unique content (i.e. being overwhelmed by others is different to the effort of putting on a false persona or from fears of saying or doing the wrong thing). The inter-item correlations for the remaining seven items ranged from .41 to .74. Alpha was .90 or ‘excellent’ [66] and corrected item-total correlations were between .63 and .83 or ‘very good’ [64].

Factor 3 alpha coefficient was α = .88 or ‘good’ [66]. Alpha was not increased by removing any item, inter-item correlations ranged from .21 to .65 and corrected item-total correlations ranged from .45 to .70 or ‘very good’ [64]. As such, no items were removed from this factor.

The remaining 33-items after these deletions formed the Experience of Time Alone Scale (ETAS), each factor constituting a subscale of the measure, as displayed in Table 6.

### Validity analyses

Content validity was built into the test from the outset, with items being derived from Study 1. Qualitative analysis of the interviews with people with BPD ensured the items reflected the experiences as described by participants. Validity was then further strengthened by the pilot studies involving individuals with BPD, healthy participants and experienced and qualified respondents. Validity of the scale was also demonstrated through significant correlations with theoretically relevant clinical measures (AMES, HEI-R, MHI-5) as displayed in Table 7.

**Table 7:** Spearman’s rho correlations of scores on the ETAS and theoretically relevant measures using BPD sample (N = 112).

	Cannot Cope Alone (Factor 1)	Need to Escape from Others (Factor 2)	Consumed in Intolerable Distress (Factor 3)	Total ETAS score
MHI-5	-.26**	-.13	-.61**	-.47**
AEMS (Aloneness)	.13	.39**	.52**	.42**
AEMS (Evocative)	.13	.18	.41**	.28**
HEI-R	.56**	.24*	.49**	.67*

Note.

*significant at less than α = 0.05

**significant at less than α = 0.01, MHI-5 = Mental Health Inventory-5, AEMS = Aloneness and Evocative Memory Scale, HEI-R = *Hurvich Experience Inventory–Revised*

As items for the scale were developed using a female-only sample, investigation into any potential gender effects was undertaken. Results revealed no statistically significant differences between the genders for any of the subscales: Cannot Cope Alone, *U* = 382, *z* = -.38, *p* = .71; Need to Escape from Others, *U* = 325.50, *z* = -1.02, *p* = .31; Consumed in Intolerable Distress, *U* = 387, *z* = -.33, *p* = .75. Total ETAS score was also non-significant across genders, *U* = 393.50, *z* = -.25, *p* = .81. However, the small sample size of men means these results should be interpreted cautiously.

The BPD scores on the ETAS were compared with the control sample scores and revealed significant differences between groups on all three subscales and the total ETAS score, as displayed in Table 8. Effect sizes of these between-group differences were estimated by Pearson’s correlation coefficient *r* [67] and were all found to be “large” [68].

**Table 8:** Comparison of BPD scores with control group on ETAS subscale and total score.

ETAS	BPD sample mean rank (*n* = 112)	Control group mean rank (*n* = 105)	*U*	*z*	*p*	Effect Size (*r*)
Cannot Cope Alone	159.64	54.99	208.5	-12.33	< .001	-.84
Need to Escape from Others	149.27	66.04	1369.5	-9.78	< .001	-.66
Consumed in Intolerable Distress	159.92	54.69	177.5	-12.35	< .001	-.84
Total ETAS	160.83	53.72	75.5	-12.56	< .001	-.85

The final version of the scale with randomized items is presented in S1 Table.

## Discussion

The aim of these studies was to develop a measure of the experience of time alone for individuals with BPD. First, Study 1 examined the essential experience of time alone for BPD participants. Based on these findings a self-report scale was created and pilot-tested to assess the soundness and clarity of the measure. Study 2 then aimed to demonstrate the reliability and validity of the developed scale through correlations with theoretically relevant measures and comparisons between groups in their responses to the scale. As hypothesized, mean scores on the ETAS and all other measures demonstrated that there were significant differences between BPD and control participants in the aversive experiences of aloneness, in general psychopathology, in the intensity of annihilation fears and in both aloneness and evocative memory. BPD participants strongly endorsed ETAS items yet control participants did not indicate that they related to or shared in these experiences to the same extent or even, sometimes, at all. Furthermore, as predicted, higher ratings of aloneness did significantly correlate to poorer general psychopathology, higher ratings of annihilation anxiety, and higher scores on existing measures of aloneness [39], including poorer evocative memory. These results suggest the ETAS to be a valid measure of the experience of time alone for people with BPD.

The accounts of a negative experience of time alone captured in Study 1 reflect previous depictions of the intolerance of aloneness in BPD. As described by previous theorists participants reported desperately trying to fill time alone with activities and by contacting others, as well as resorting to maladaptive means such as self-harm to rid themselves of their overwhelming distress and rage [33, 34]. These findings are consistent with recent research into the separate but related construct of chronic emptiness, in which chronic feelings of emptiness were significantly related to self-harm behaviours for individuals with BPD [69]. It would be expected that these chronic feelings would be experienced as more intense in the context of being alone. Indeed, Köhling et al. [70] report that for individuals with major depressive disorder and BPD, significant reductions in mood were reported after a period of being alone. This reduction in mood was significantly more intense than a comparison group of individuals with major depression only, indicating that a lower tolerance for being alone may be specific to BPD.

Pazzagli and Monti [35] argue that in line with Kernberg’s [71, 72] pain-anger-hate-vengefulness cycle this rage may function to punish yet preserve a sense of an absent other. Rumination, worry and negative thinking may be other ways individuals preserve a sense of the other in their absence, and participants reported that it was the intensity and intrusiveness of their negative thoughts that made time alone intolerable. However, persistent rumination (as opposed to escalating distress and emotion) and the experience of being bombarded by intrusive negative thoughts has not been the focus of previous descriptions of aloneness. Similarly, theories have not tended to focus on individuals difficulties initiating activity via indecision and a pattern of becoming lethargic, depressed, depleted and isolating themselves away from others. This tendency to sleep or become completely inactive was reported as lasting for days or weeks on end, and may represent Masterson’s [18, 73] abandonment depression, an underlying anaclitic depression [74] or the core of aloneness that Gunderson and Links [36] suggest can result in psychotic symptoms and dissociation.

A further significance of Study 1 is that participants described actually craving time alone, an aspect that is missing from many accounts of aloneness in BPD. This positive experience was not due to feelings of contentment and peace when alone, but rather, because of the relief experienced when escaping the demands of relating, supporting the idea that alone time can be conceptualized as a need [75, 76]. Together, the positive and negative experiences of time alone can be understood as consistent with Dazzi’s [8] view that both intolerance of aloneness and intolerance of relating are pivotal in BPD. Individuals reported struggling with feelings of alienation, and would hide themselves with a false persona out of fears of rejection [16, 77]. Additionally, fears of intrusion and engulfment preoccupied individuals when around others.

In Study 2, group comparisons revealed significant differences between control and BPD participants in all demographic comparisons, many of which were unsurprising given the nature of the diagnosis and the psychosocial impact of having a serious mental illness. As such these differences were not anticipated to impact meaningfully on the validity of results. Differences between groups on all measures were significant indicating those with BPD were more distressed (MHI-5), had more severe problems with aloneness and evocative memory (AEMS), and experienced more annihilation anxiety (HEI-R) compared to non-BPD controls.

Results from the between-groups comparison also showed that the ETAS clearly taps into the borderline experience of aloneness, with very large differences between the individuals with BPD and controls on all three subscales and the total score. The three factors that make up the ETAS reflect theoretically relevant areas of concern for individuals with BPD that healthy controls do not tend to struggle with. The ‘Cannot Cope Alone’ factor encapsulates notions of dependency, fears of abandonment as consistent with DSM-5 criteria [1] and is similar to a presentation of preoccupied attachment in BPD [78]. The ‘Need to Escape from Others’ factor depicts a fear of being judged, being intruded upon and rising irritability around others. This factor may be understood by the notion of an ‘alien self’ [31], or of hyper-mentalization, reflecting a consuming preoccupation with the assumed thoughts and feelings of others as well as an automatically negative self-appraisal [79]. It may also reflect annihilation from a weak self being encroached upon by external demands and stimuli [80]. As such, time alone is felt as a relief from the effortful nature of interacting. The ‘Consumed in Intolerable Distress’ factor most clearly resembles Fromm-Reichmann’s [25] description of aloneness, as being unable to recall a soothing presence, and thus left helpless with annihilating feelings and consuming negative mental states.

Finally, validity of the ETAS was also demonstrated through significant correlations with theoretically relevant measures reflecting that, as expected, individuals with more severe experiences of time alone have greater distress, increased difficulties with both aloneness and evocative memory, and stronger reported feelings of annihilation.

There are two caveats to consider with respect to these studies. First, participants in Study 1, which formed the basis of the ETAS, were all women. While further examination of differences between genders on scores of the ETAS revealed no significant differences, the small sample size of men means these results should be interpreted with caution. It may also indicate that there are core themes of the experience of time alone that men encounter that are not reflected in the scale items. Qualitative interviews with males diagnosed with BPD investigating their experience would be a possible avenue for future research.

The second caveat is that an online format was used for administering the scale in Study 2, which prevents assessing BPD presentation via a formal structured clinical interview and potentially impacts validity and generalizability of results. However, data collection via online platforms has previously been found to be effective and reliable, particularly for personality disorder research [52]. Further, as results demonstrate clear discrimination between BPD and control participants, this indicates that this method is adequate and that the measure was sensitive. However, as the reliability and preliminary validity was established in an online format, it needs to be recognised that findings may not be generalisable to paper and pencil versions of the scale, or versions of the scale conducted in group or interpersonal settings. Whilst there is no a priori reason that results in different contexts or formats would be different, this is something that has not been established with the ETAS and could be a direction for future research.

Given BPD participants’ scores were so clearly different to control scores, and given fear and intolerance of being alone is linked to most, if not all, current DSM-5 criteria [36], the ETAS has clinical potential as a diagnostic aide. The scale also demonstrates clinical utility through various individual items that would prove useful in a therapy setting. For instance, endorsement of items such as ‘I get so upset when I am alone that I hurt myself’, and ‘I sleep to avoid being alone’ would possibly warrant further exploration in psychotherapy, and as such, use of the ETAS may enhance and guide treatment. Future studies utilizing a large cohort of representative participants could assess the robustness of these findings, could further investigate the relationship between aloneness and BPD diagnosis, and could explore interactions with self-harming or suicidal behaviors. Further, while these studies focused on aloneness in the context of a specific BPD population, the current conversation regarding dimensional versus categorical approaches [9, 81] may indicate the experience of being alone as a relevant feature for a wide variety of severe personality (or other) pathology. As such, investigating the experience of being alone within a transdiagnostic or dimensional trait based framework such as the borderline personality ‘organisation’ [82] or borderline ‘pattern’ [83] would be of interest. Alternatively, if a categorical approach is favored, exploring the experience of time alone for other personality disorders (e.g. narcissistic, dependent) may also be an avenue for future research.

The ETAS is the only measure that assesses the experience of time alone in BPD as described by individuals with the disorder. Time alone for people with BPD is a psychological challenge and, along with the experience of relating, warrants renewed attention. As such, this measure provides clinicians with an evidence-based instrument to assist the treatment process, responding to the need for continued focus on effective treatment and research into BPD [4, 84].

## Supporting information

S1 Table Experience of time alone scale. (DOCX)
