# Supplementary material for: Development and validation of an experience of time alone scale for borderline personality disorder
Source: PLoS One. 2019 May 23;14(5):e0217350. doi: 10.1371/journal.pone.0217350 (PMC6532922; doi:10.1371/journal.pone.0217350)
Supplement: S1 Table — (DOCX) [file pone.0217350.s001.docx]

Supplementary materials

Experience of Time Alone Scale

|  | Not at all | A little bit | A moderate amount | A great deal |
| --- | --- | --- | --- | --- |
| 1. When I am alone I dwell on things I have done wrong | 1 | 2 | 3 | 4 |
| 1. When I am alone I wish someone was with me to help me feel OK | 1 | 2 | 3 | 4 |
| 1. Time alone is relief from the effort it takes to relate to others | 1 | 2 | 3 | 4 |
| 1. When I am alone I feel so abandoned I will desperately seek contact with other people | 1 | 2 | 3 | 4 |
| 1. I need to have time alone because I get irritable and edgy around others | 1 | 2 | 3 | 4 |
| 1. When I am alone I enjoy pampering and doing nice things for myself | 1 | 2 | 3 | 4 |
| 1. When I am alone I cannot work out what to do with myself | 1 | 2 | 3 | 4 |
| 1. When I am alone my mind becomes filled with negative thoughts about the past | 1 | 2 | 3 | 4 |
| 1. When I am alone I wish someone was there to motivate me | 1 | 2 | 3 | 4 |
| 1. I need time alone to escape from the pressure of other people’s expectations | 1 | 2 | 3 | 4 |
| 1. When I am alone I think more about suicide | 1 | 2 | 3 | 4 |
| 1. When I am alone I wish someone was there to tell me what to do | 1 | 2 | 3 | 4 |
| 1. When I am alone I feel motivated to do things that I enjoy | 1 | 2 | 3 | 4 |
| 1. When I am alone I am very critical of myself | 1 | 2 | 3 | 4 |
| 1. I need to escape and be by myself to avoid being totally overwhelmed by others | 1 | 2 | 3 | 4 |
| 1. Time alone is freedom from the worry that I will say or do the wrong thing around others | 1 | 2 | 3 | 4 |
| 1. Alone I feel overwhelmed by simple tasks and have to push myself to do them | 1 | 2 | 3 | 4 |
| 1. I get so upset when I am alone that I hurt myself | 1 | 2 | 3 | 4 |
| 1. When I am alone I still know that people care | 1 | 2 | 3 | 4 |
| 1. Silence is scary when I am alone | 1 | 2 | 3 | 4 |
| 1. I sleep to avoid being alone | 1 | 2 | 3 | 4 |
| 1. I can feel a sense of inner peace and contentment when I am alone | 1 | 2 | 3 | 4 |
| 1. I seek out others to avoid being alone | 1 | 2 | 3 | 4 |
| 1. I need medication or alcohol/drugs to help me cope with my distress when I am alone | 1 | 2 | 3 | 4 |
| 1. It is a relief to be alone because I do not have to maintain a false self or mask to conceal the real me | 1 | 2 | 3 | 4 |
| 1. I feel hopeless about my life when I am alone | 1 | 2 | 3 | 4 |
| 1. When I am alone I get full of rage | 1 | 2 | 3 | 4 |
| 1. When I am alone I feel lonely and wish for company | 1 | 2 | 3 | 4 |
| 1. When I am alone I still feel my life has meaning and purpose | 1 | 2 | 3 | 4 |
| 1. I stay distressed alone until someone else can help me feel better | 1 | 2 | 3 | 4 |
| 1. Alone I can choose not to think about issues that are bothering me and get on with other things | 1 | 2 | 3 | 4 |
| 1. I avoid being around others because I feel like I do not fit in | 1 | 2 | 3 | 4 |
| 1. I feel myself getting anxious when I am alone | 1 | 2 | 3 | 4 |

*Note*. Items 6, 13, 19, 22, 29 and 31 are reverse scored

Suppressed and excluded items from scale

| Item | Reason for removal |
| --- | --- |
| I need time alone to unwind and de-stress | Control score > than BPD score |
| I need the TV, radio or music on to fill the silence when I am alone | Factor 1 loading < .5 |
| When I am alone I worry that I am not thinking right | Factor 1 loading < .5 |
| I cannot settle into an activity when I am alone | Factor 1 loading < .5 |
| When I am alone my mind is so busy it does not stop | Factor 1 loading < .5 |
| I dissociate/space out to avoid time alone | Factor 1 loading < .5 |
| When I am alone I use drugs or alcohol to escape for a while | Factor 1 loading < .5 |
| I can enjoy doing activities by myself | Factor 1 loading < .5 |
| To cope alone I have to keep myself busy with activities | Factor 1 loading < .5 |
| When I am alone I eat too much | Factor 1 loading < .5 |
| When I am alone I enjoy the chance to relax and be at peace | Factor 2 loading < .5 |
| When I am alone I enjoy the freedom to do what I want, when I want | Factor 2 loading < .5 |
| When I am alone I stress about my interactions with others | Factor 2 loading < .5 |
| I need time alone because I sacrifice my needs around others | Factor 2 loading < .5 |
| When I am alone I structure my day so I am not left doing nothing | Factor 3 loading < .5 |
| When I am alone I argue and battle with myself in my head | Factor 3 loading < .5 |
| Alone my mood spirals downwards and I can not stop it | Factor 3 loading < .5 |
| Alone I isolate and hide away from the world | Factor 3 loading < .5 |
| Alone I am able to calm myself down if I am upset | Factor 3 loading < .5 |
| When I am alone I hear voices inside my head | Factor 3 loading < .5 |
| When I am alone I sit and do nothing for hours | Factor 3 loading < .5 |
| When I’m alone I crave a deep personal connection with someone | Inter-item correlation < .2 |
| I need time alone to work out how I feel about things | Alpha increased if removed |
| It is a relief to be on my own because I find it too intense around others | Inter-item correlation > .7 |
| Time alone is a relief from feeling self-conscious around others | Inter-item correlation > .7 |
